# A Modified Sparse Representation Method for Facial Expression Recognition

**DOI:** 10.1155/2016/5687602

**Published:** 2016-01-04

**Authors:** Wei Wang, LiHong Xu

**Affiliations:** Department of Control Science and Engineering, School of Electronics and Information Engineering, Tongji University, Shanghai 200092, China

## Abstract

In this paper, we carry on research on a facial expression recognition method, which is based on modified sparse representation recognition (MSRR) method. On the first stage, we use Haar-like+LPP to extract feature and reduce dimension. On the second stage, we adopt LC-K-SVD (Label Consistent K-SVD) method to train the dictionary, instead of adopting directly the dictionary from samples, and add block dictionary training into the training process. On the third stage, stOMP (stagewise orthogonal matching pursuit) method is used to speed up the convergence of OMP (orthogonal matching pursuit). Besides, a dynamic regularization factor is added to iteration process to suppress noises and enhance accuracy. We verify the proposed method from the aspect of training samples, dimension, feature extraction and dimension reduction methods and noises in self-built database and Japan's JAFFE and CMU's CK database. Further, we compare this sparse method with classic SVM and RVM and analyze the recognition effect and time efficiency. The result of simulation experiment has shown that the coefficient of MSRR method contains classifying information, which is capable of improving the computing speed and achieving a satisfying recognition result.

## 1. Introduction 

Facial expression is an important way of nonverbal communication [1], which cannot only reflect the inner world of human beings but also occupy a very important position in human communication. Facial expression recognition relates to graph pattern recognition, image processing, computer vision, cognitive science, psychology, physiology, and other disciplines [2–4]. Understanding and research of facial expression recognition will promote the development of these related disciplines. Face expression recognition technology has penetrated into many areas of daily life.

In the field of image processing, Candes and Wakin indicate that the recovery process of the original image is an optimization problem [5]. Compressed sensing can carry on sampling and compression of the image simultaneously at a low rate, greatly reducing the cost of sampling and calculation. Therefore, compressed sensing is widely used in the image processing, where sparse solutions used for facial image identification or expression classification are a new direction in related fields. The main idea of this method is the use of a linear combination of a number of training samples to represent the test sample and achieve recognition and classification according to the sparse representation. In this way, the vast majority of the sparse representation coefficient in the training samples involved in the reconstruction is close to zero or zero. An excellent recognition system is to have a good test face to find unique projection in face training samples and has good robustness to noise. Current research on this field has put out some valuable studies. Wright et al. applied compressed sensing based sparse representation theory to face recognition [6]. The robustness of sparse representation classification (SRC) was better than commonly used classification methods. Zhang and Li proposed a face recognition method based on dictionary learning [7], constantly updating optimized dictionary, so that it contained classified information more effectively. In recent years, many researchers have applied the sparse representation theory to expression recognition. Cotter used SRC to major organs of face and evaluated the classification performance of various parts and made integration of the results [8]. For large shielding area of test images, the recognition effect was still in an acceptable range. Zhang et al. adopted Gabor filter to extract feature and carried on expression classification with SRC [9] and compared the proposed method with SVM, NN, and other methods. Zhi et al. transformed the problem of facial expression recognition to minimized *l*_1_ norm and combine with the fuzzy integral method [10]. Simulation results showed that the method could achieve good frontal facial expression recognition and had robustness to shadowed face. El-Sayed et al. combined multi-Gabor filters with sparse representation for feature extraction [11] and used SVM classifier to identify different expressions, obtaining a satisfactory recognition rate. Some expression recognition studies focus on the combination of feature extraction method and SRC, and the difference mainly lies in the use of feature extraction methods [12, 13]. In [12], Mohammadi et al. used classical PCA based dictionary to achieve accurate facial expression classification by sparse representation. The experimental results showed that the recognition rate using this framework was 6% higher than other methods and was expected to be used for target recognition. In [13], Wang and Ying proposed a sparse representation method based on Gabor feature extraction and Adaboost selection for facial expression recognition. In JAFFE database, the authors compared the method with classic methods such as 2DPCA+SVM and LDA+SVM. The result verified the higher recognition rate of the proposed method. There are also some studies on the training sample based overcomplete dictionary updating, with the purpose of enhancing the ability of dictionary expression and improving recognition efficiency of SRC [14, 15].

In this paper, we study an expression recognition method by a sparse representation method. Firstly, we use Haar-like+LPP [16] to extract feature and reduce dimension. Add block dictionary training mode to LC-K-SVD instead of adopting directly the dictionary from samples. Then, use stOMP on the classification stage to speed up the convergence rate of the traditional OMP and a dynamic regularization factor to suppress noises and enhance accuracy. Finally, this method is used in different facial expression databases for comparison with different algorithms from different aspects. Experimental results show that the proposed sparse representation method can be applied to facial expression analysis and has its own advantages in certain aspects.

## 2. Dictionary Learning Classification Algorithm

This paper uses sparse representation algorithm for facial expression recognition and is divided into two steps [18]: dictionary learning and algorithm classification.

The problem of sparse solution is as follows:

(1) $$\begin{array}{c} \langle \;W,H\;\rangle =\underset{\langle W,H\rangle }{\min}{\left\|\;V-WH\;\right\|}_{2}^{2}+\lambda \underset{i,j}{\sum }\left|\;H_{i,j}\;\right|, \end{array}$$

where **W** represents the coefficient matrix of linear classifier, **H** = [*h*_1_,…, *h*_*N*_] ∈ **R** ^*m*×*N*^ is the label of the input signal, and *h*_*i*_ = [*h*_*i*_^1^, *h*_*i*_^2^,…,*h*_*i*_^*k*^]^T^ = [0,…, 0,1, 0,…, 0] ∈ **R** ^*m*^, *h*_*i*_^*k*^ = 1, represent that the corresponding label of input signal *x*_*i*_ is *k*. *λ* is the sparse adjustment coefficient; the higher *λ* is, the greater the sparse constraint is and the sparser the coding is. ∑_*i*,*j*_|**H**_*i*,*j*_| is the *l*_1_ norm of sparse matrix **H**.

In facial expression classification, we can use the entire training set as a dictionary. However, the classification efficiency will get lower with the increase of training data. It is sometimes necessary to preprocess the training set data before the classification, such as getting an abstract dictionary by certain dictionary learning method. Dictionary learning is an important part of sparse representation classification. The formula of dictionary learning is as follows [10]:

(2) minD∈C⁡fnD=minD∈C⁡1n∑i=1nIxi,D,Ixi,D=minα∈Rk⁡12x−Dα22+λα1.

In formula (2), *n* is the number of faces in training set. *α* is sparse coefficients of face *x* in dictionary **D**. Search sparse coefficient *α* on the basis of **D**, and update dictionary **D** on the basis of *α*. Final dictionary **D** is got by continuous iterations. Common dictionary learning algorithms include LC-K-SVD [18] and Online Dictionary Learning [19].

Given a test face expression *y*, find the sparse coefficient ∂ in dictionary **D**, so as to make *y* ≈ **D**∂. For each class, calculate the reconstruction error, respectively. The class with minimum error corresponds to the class *y*:

(3) yi≈D∂i,classy=arg miniy−yi,

where ∂_*i*_ is the sparse component of the *i*th class, *y*_*i*_ is the reconstructed face of the *i*th class, and class(*y*) is the class of test data *y*.

**Figure 1:**
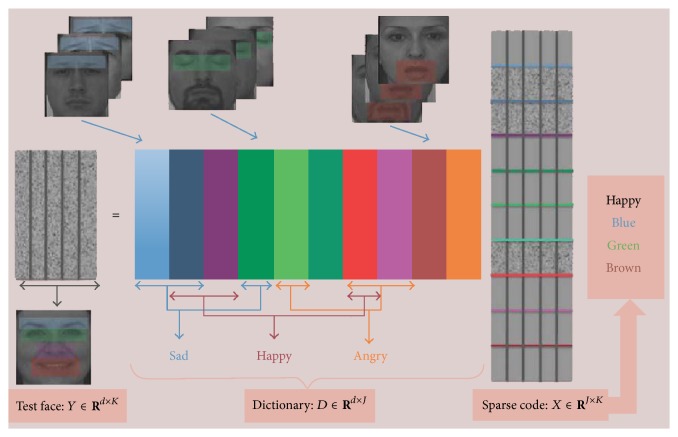
Frame of sparse expression representation by dictionary learning [17].

The scheme of dictionary learning based sparse expression classification is presented in Figure 1.

## 3. Modified Sparse Representation Recognition Method

In this section, we will show the modified sparse representation recognition based on block dictionary learning (LC-LSVD) and fast classification (stOMP) method.

Label consistency based K-SVD dictionary learning algorithm LC-K-SVD [18] is one of the sparse dictionary learning methods. Its central idea is to add label consistency constraint when solving the model, using K-SVD iterative learning algorithm, to achieve a linear classifier:

(4) D,A,X=arg⁡minD,A,X⁡Y−DX22+αQ−AX22s.t.  ∀i,  xi0≤e,

where **D** is a dictionary matrix, the goal of learning. **Y** is a training sample set and **X** is sparse solution, code for **Y** learned in the dictionary **D**. **A** represents a linear transformation matrix and linear transformation *g*(*x*; **A**) = **A** *x* makes original sparse representation more discriminative in sparse space. One has

(5) $$\begin{array}{c} Q=\left[\begin{array}{cccccccc} 1 & 1 & \cdots & 0 & 0 & \cdots & 0 & 0\\ 0 & 0 & \cdots & 1 & 1 & \cdots & 0 & 0\\ \cdots & \cdots & \cdots & \cdots & \cdots & \cdots & \cdots & \cdots \\ \cdots & \cdots & \cdots & \cdots & \cdots & \cdots & \cdots & \cdots \\ \cdots & \cdots & \cdots & \cdots & \cdots & \cdots & \cdots & \cdots \\ 0 & 0 & \cdots & 0 & 0 & \cdots & 1 & 1 \end{array}\right], \end{array}$$

which is discriminative sparse coding, each column of which represents an image. Nonzero elements represent the class of the image, used to constrain the sparse solution **X**, making the sparse solution of the same class more consistent. When **D**_*i*_ (*i* represents the *i*th class) and **Y**_*i*_ represent the same class, **Q** is 1 and 0, otherwise. ‖**Y** − **D** **X**‖_2_^2^ is reconstruction error between sparse representation and the original samples. *α*‖**Q** − **A** **X**‖_2_^2^ is a label consistency constraint, indicating the label errors. *α* represents the adjustment coefficient.

**Figure 2:**
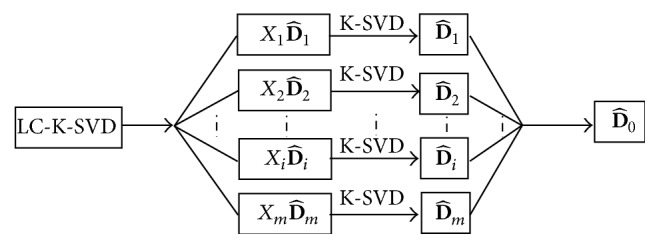
Flow chart of initialization dictionary within class in LC-K-SVD [18], where *m* represents the number of labels (*k*).

For algorithm initialization, we need to initialize dictionary **D**_0_ and a linear transformation matrix **A**_0_. Randomly select a part of samples from each class, to form the label initialized matrix ${\hat{D}}_{i}$. First of all, train the subdictionary ${\hat{D}}_{i}$ in class. Then, cascade all kinds of training dictionary ${\hat{D}}_{i}$ to be initialized dictionary ${\hat{D}}_{0}=[{\hat{D}}_{1},{\hat{D}}_{2},\ldots ,{\hat{D}}_{m}]$. After training, various types of samples in ${\hat{D}}_{0}$ retain the basic features of each type of sample, reducing the error of initialized dictionary. The idea of dictionary training is shown in Figure 2.

Using multiple and ridge regression model to solve **A**,

(6) $$\begin{array}{c} A=\arg\underset{A}{\min}{\left\|\;Q-AX\;\right\|}^{2}+\lambda {\left\|\;A\;\right\|}_{2}^{2}. \end{array}$$

**A** = (**X** **X** ^T^ + *λ* **I**)^−1^ **X** **Q** ^T^, where **X** is sparse representation matrix.

Then, use K-SVD learning algorithm:

(7) D,A,X=arg⁡minD,A,X⁡YαQ−DαAX22s.t.  ∀i,  xi0≤e.

Set $Y_{new}=(Y^{T},\sqrt{\alpha }Q^{T})$, $D_{new}=(D^{T},\sqrt{\alpha }A^{T})$, and then formula (7) is transferred to

(8) Dnew,X=argminDnew,X⁡Ynew−DnewX22s.t.  ∀i,  xi0≤e.

**D**_new_ is solved by the K-SVD algorithm iterations and transferred to dictionary **D** and linear transformation matrix **A**. The transforming process is as follows, where *k* represents the number of labels:

(9) D^=d1d12,d2d22,…,dkdk2,A^=a1a12,a2a22,…,akak2.

For the test face *y*, we solve the sparse coding value *x* by a sparse method called orthogonal matching pursuit algorithm. Orthogonal matching pursuit algorithm [20], developed as an improvement to matching pursuit, shares many properties of matching pursuit. In each iteration, orthogonal matching pursuit calculates a new signal approximation. The approximation error is then used in the next iteration to determine which new element is to be selected. In particular, the selection is based on the inner products between the current residual and the column vectors of the dictionary. On the classification stage of sparse solution, we use OMP based method to solve the problem of facial expression:

(10) $$\begin{array}{c} \underset{x}{\min}{\left\|\;y-Dx\;\right\|}_{2}+\gamma {\left\|\;x\;\right\|}_{p}, \end{array}$$

where** D** is the optimal dictionary by LC-K-SVD dictionary training method. *γ* is a regularization parameter, is of control of the sparsity, and rejects noises in certain degree. The norm *l*_*p*_ is a quantitative index to measure the sparsity of a signal. When *p* = 0, it means that we search minimum number of nonzero components. It is usually stated that searching the minimum *l*_0_ norm is an intractable problem as the dimension increases (because it requires a combinatorial search) and as the noise increases (because any small amount of noise completely changes the *l*_0_ norm of a vector). When *p* = 2, *l*_2_ norm is the convex approximation of *l*_0_ norm; therefore, it is not sparse. When *p* = 1, *l*_1_ norm is the convex approximation of *l*_0_ norm, as well as the concave approximation of *l*_0_ norm; therefore, it is sparse. In this paper, we set *p* = 0.5 (empiric value between 0 ≤ *p* ≤ 1), when we can achieve the best sparse solution and reconstruction effect.

The optimization problem (10) is concave when *p* ≤ 1, so there will be multiple local minima. The OMP algorithm is only guaranteed to converge to one of these local minima. The algorithm is sensitive to initial conditions and prior information may be incorporated into the initialization to help converge to the global solution. An efficient way is adjusting the regularization parameter *γ* and reinitialization to escape from local optima.

The optimum *γ* can be determined by parameter regularizing criteria. Usually the higher the noise power is, the larger *γ* is. For the regularization parameter *γ*, a number of methods have been proposed, including quality-of-fit, sparsity, and the L-curve [21]. Here we adopt a heuristic method that allows the tradeoff between error and sparsity to be tuned for each iteration [22, 23],

(11) $$\begin{array}{c} \gamma =\gamma_{max}\left(\;1-\frac{\left\|y-D\hat{x}\right\|}{\left\|y\right\|}\;\right),\;\gamma >0, \end{array}$$

where *γ* is a heuristic regularization term, limited by *γ*_max_ which controls the tradeoff between sparsity and reconstruction error. Commonly, higher values of *γ* lead to more sparse solutions, at the cost of increased error. We can set *γ*_max_ = SNR^−1^ if the signal-to-noise ratio (SNR) can be estimated.

In OMP, only a single element is selected in each iteration, so that the algorithm has to be run many iterations as there are nonzero elements to be estimated. This can only be avoided in a greedy algorithm by selecting more than a single element in each iteration. Here we adopt Stagewise OMP (StOMP) [24] method. It calculates a threshold *λ* and then selects all elements, whose inner products have a magnitude larger than the threshold:

(12) $$\begin{array}{c} \lambda =\frac{t{\left\|r^{n-1}\right\|}_{2}}{\sqrt{M}}, \end{array}$$

where *n* represents the *n*th iteration. *M* is the number of rows in an overcomplete matrix. The choice of parameter *t* will affect the performance of the algorithm, but no specific selection method can be adopted. Choose the atomic of **D** that the inner product is greater than the threshold value:

(13) $$\begin{array}{c} \Gamma^{n}=\Gamma^{n-1}\cup \left\{\;i:\left|\;\alpha_{i}\;\right|\geq \lambda \;\right\}, \end{array}$$

where **α**_*i*_ is the inner products between the current residual $r^{n}=y-{\hat{y}}^{n}$ and the column vectors **D**_*i*_ of the dictionary **D**.

**Figure 3:**
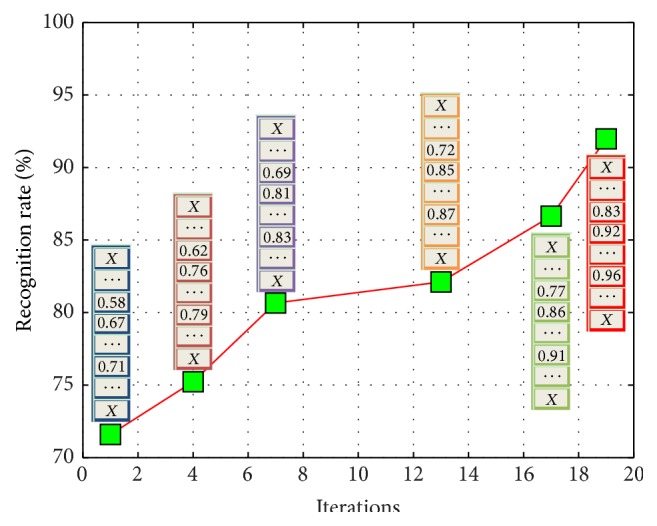
Recognition rate of the sparse classifier increases as iteration goes. A vector is used to store the recognition rates of all features, where “*X*” means that the feature is not selected.

The iteration process of stOMP based sparse expression classification is shown in Figure 3.

**Figure 4:**
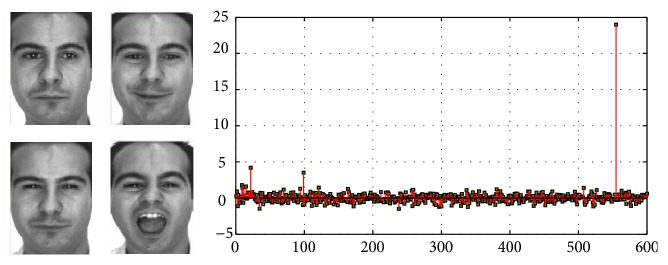
Diagram of coding distribution (dataset is Extended Yale B, encoding for the face on the left, coding for the training process) [18].

Using multiple and ridge regression model to get coefficient matrix of linear classifier **W**, **W** = (**X** **X** ^T^ + *λ* **I**)^−1^ **X** **H** ^T^. Then we use a linear classifier, as shown in formula (14). The diagrammatic sketch of the coding distribution by our algorithm is shown in Figure 4. Consider

(14) $$\begin{array}{c} j=\arg\max\left(\;l=Wx_{i}\;\right). \end{array}$$

In formula (14), *l* is a vector and sparse coding *x*_*i*_ can be viewed as the weight of each atomic (column) for reconstructed test image; therefore, we can regard each column of **W** as the similarity with each column of **D**. *l* = **W** *x*_*i*_ can be seen as the weight of similarity between test image *y*_*i*_ and each class. One has *l* = {0,0,…, 1,…, 0,0}. There is only one nonzero entry in *l*, that is, 1. The location of this nonzero entry determines the final expression recognition class.

In this paper, we take the use of block dictionary learning LC-K-SVD algorithm to build up overcomplete dictionary and then use stOMP algorithm to carry on classification process, in order to accelerate the speed of traditional OMP algorithm, combined with antinoise factor. To be convenience, the proposed sparse expression recognition classifier in this paper is renamed MSRR (modified sparse representation recognition). Basic sparse representation, without a dictionary to learn, using OMP method, is named SRC (sparse representation classification).

**Algorithm 1 (modified sparse representation recognition method).**

(*Contribution*: Haar-like+LPP feature extraction and dimension reduction, LC-K-SVD block dictionary learning, dynamic regularization factor, different selection vector strategy per iteration).

*LC-K-SVD Dictionary Learning Process*

*Input*. Training images with corresponding expression labels **Y** = {*y*_*i*_}_*i*=1_^*m*×*n*^, original dictionary **D**, column label matrix **H**, Discrimination sparse coding **Q**, Sparse threshold *e*.

*Output*. Final dictionary **D**, linear transformer matrix **A**, classification parameter **W**(*m* × *n*), sparse solution **X**(*n* × 1).

*Preprocessing*. Feature extraction and dimension reduction by Haar-like+LPP method. The size of preprocessed image is *m* × 1.

Procedure:

(1) Initialize: dictionary **D** ^0^, linear transformation matrix **A** ^0^, *j* = 1.
(2) Sparse coding:
  (15) D,A,X=arg⁡minD,A,X⁡Y−DX22+αQ−AX22,s.t.  ∀i,  xi0≤e.
(3) Dictionary updating stage:
 Set $Y_{new}=(Y^{T},\sqrt{\alpha }Q^{T})$, $D_{new}=(D^{T},\sqrt{\alpha }A^{T})$.
 K-SVD Dictionary updating: Trained dictionary **D** is achieved by LC-K-SVD method
  (16) Dnew,X=arg⁡minDnew,X⁡Ynew−DnewX22s.t.  ∀i,  xi0≤eD=Dnew1:m,1:k∗n,A=1αDnewm+1:m+k,1:k∗n.
(4) End: The change of ‖**Y** − **D** **X**‖_2_^2^ is less enough; or, *j* = *j* + 1, go to Step (2).

*Discrimination Classification Stage*

*Input.* Test sample image *y*, trained dictionary** D** by LC-K-SVD.

*Output.* Label *j* of test sample image.

Procedure:

(1) Calculate the sparse coding *x* for a test sample image *y*.
 Object: min_*x*_⁡‖*y* − **D** *x*‖_2_ + *γ*‖*x*‖_*p*_.
 Initialize: Set *n* = 1, iteration termination error *ε* and the maximum iteration number  *T*_max_, initialize distribution of sparse solution *x* ^0^ = 0, *r* ^0^ = *y*, Γ^0^ = *∅*.
(2) For *n* = 1; *n*≔*n* + 1 till stopping criterion is met.
(3) *i* ∉ Γ^*n*−1^, *α*_*i*_ = **D**_*i*_^T^ **r** ^*n*−1^.
(4) Selecting more than a single element in each iteration: $\left|\alpha_{i}\right|\geq t{\left\|r^{n-1}\right\|}_{2}/\sqrt{M}$.
(5) $\Gamma^{n}=\Gamma^{n-1}\cup \left\{i:\left|\alpha_{i}\right|\geq t{\left\|r^{n-1}\right\|}_{2}/\sqrt{M}\right\}$.
(6) Update distribution of sparse solution:
  (17) xΓnn=DΓn+γnI+yγn=γmaxn1−y−DΓnxΓnny,γn>0.
(7) **r** ^*n*^ = *y* − **D** *x* ^*n*^.
(8) Judge termination condition. Comparing the difference between the prior and the last distribution of sparse solution, if ‖*x* ^*n*^ − *x* ^*n*−1^‖ or *n* ≥ *T*_max_, terminate the iteration and *x* ^*n*^ is the final distribution of sparse solution; else *n* = *n* + 1, and jump to Step (4) and go on.
(9) Output sparse solution *x*.
(10) Linear classifier: *j* = argmax⁡(*l* = **W** *x*), where, the location of non-zero entry in *l* determines the final expression recognition class.

## 4. Simulation Results and Analysis

**Figure 5:**
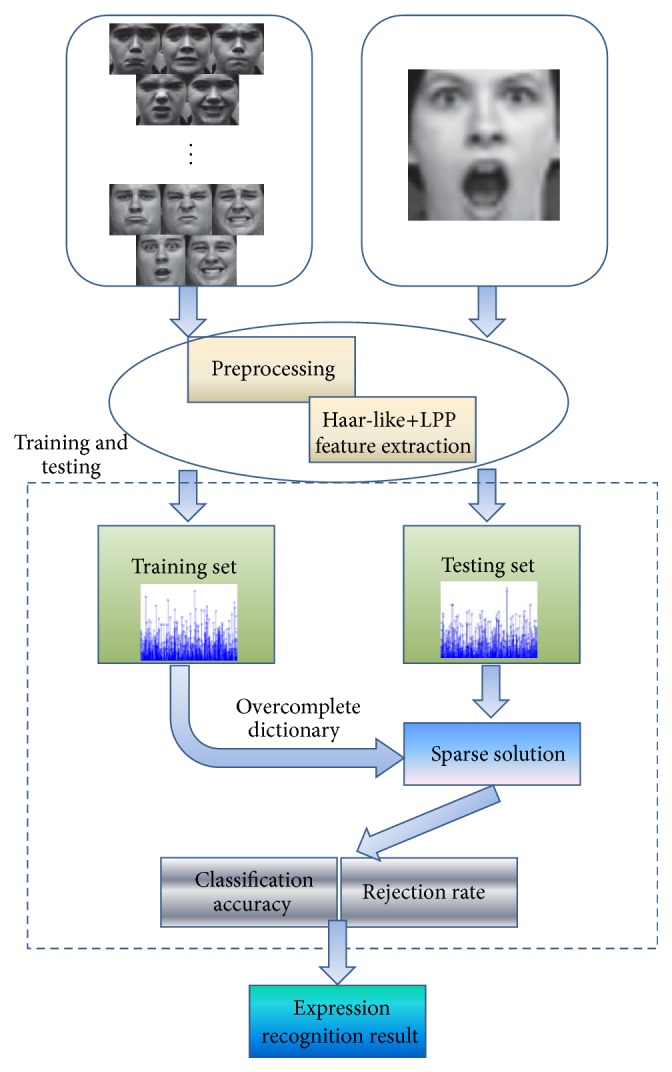
Diagram of facial expression recognition based on our modified sparse method.

The diagram of our modified facial expression recognition is shown in Figure 5.

In this section, we will validate the classification performance of the proposed sparse representation algorithm on experimental level. In typical and self-built database (infants and children expression database, JAFFE database [25], and Cohn-Kanade database [26]), we select samples for training and testing, to verify the feasibility and effectiveness of the proposed sparse representation classification method from the perspective of training samples, feature dimension, feature extraction, and dimensional reduction and noise sensitivity [27]. Further, our method is compared with SVM and RVM to analyze the effect and time complexity of the recognition algorithm.

### 4.1. Comparison of Different Training Samples and Feature Dimensions

**Figure 6:**
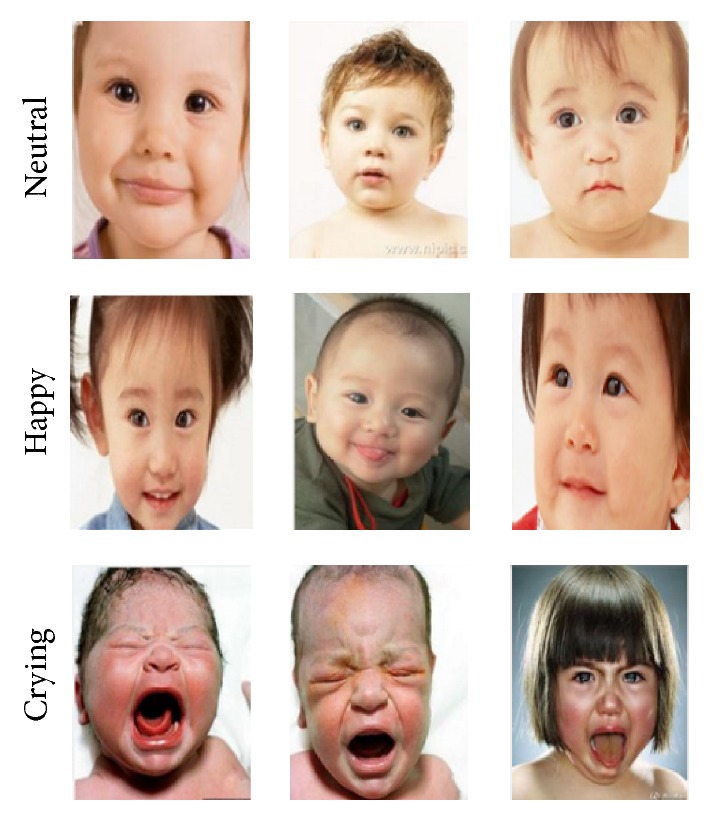
Part of the images in self-built infant and children expression database.

The self-built infant and children expression database we use is originated from the internet and preprocessed. The total number of the collected images is 900, 300 for each class: neutral, happy, and crying.  Figure 6 shows part of the images in self-built infant and children expression database.

**Figure 7:**
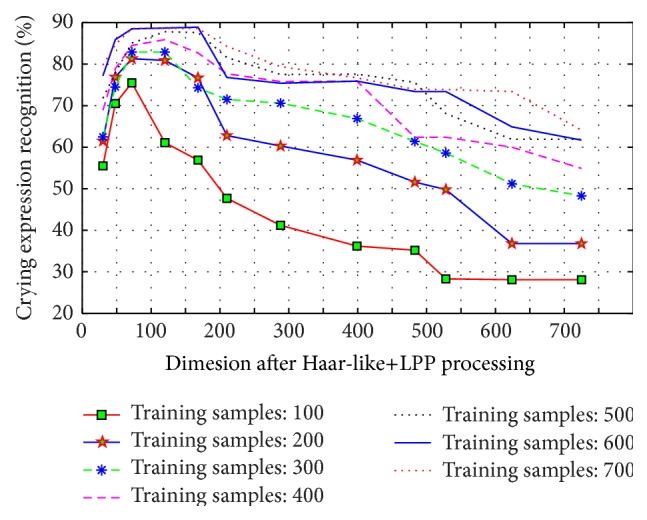
Chart of crying expression recognition with different number of training samples and feature dimension.

In this part of the experiment, the images are limited in number. Therefore, in order to ensure the universality of the experimental results, we take 100, 200, 300, 400, 500, 600, and 700 images as training samples, respectively, and adopt the LOO (leave one out) cross-validation approach in each experiments. Use Haar-like+LPP method for feature extraction and dimension reduction and select dimensions 30, 48, 72, 120, 168, 210, 288, 399, 483, 528, 624, and 725 for sparse representation classification and recognition.  Figure 7 shows the recognition rate for different training samples and dimensions of the crying expression.

We can see from the experiment that with the increase of training samples, the classification accuracy of the test sample is also gradually increased. When the test samples reach 600 and 700, the correct classification rate is at a higher level. That means the training sample 600 is sufficient for this experiment. When the training sample is 700 and the feature space dimension reaches 725, the recognition rate drastically reduces. When the training sample is 600 and the feature space dimension reaches 624, the recognition rate drastically reduces. When the training sample is 500 and the feature space dimension reaches 528, the recognition rate drastically reduces. When the training sample is 400 and the feature space dimension reaches 483, the recognition rate drastically reduces. When the training sample is 300 and the feature space dimension reaches 399, we can get similar result. When the training sample is 200 and the feature space dimension reaches 218, the recognition rate drastically reduces. When the training sample is 100 and the feature space dimension reaches 120, we will face the same problem.

We analyze the mathematical model: each column in matrix **D** is obtained by feature images of training samples, where the number of columns *n* represents the number of training sample images and the number of rows *m* represents the dimension per image. The sparse representation of test sample *y* is decided by sparse solution *x*. By solving the equation *y* = **D** *x*, we can get information about the test sample of *y*. Since the number of training samples *n* is greater than the number of the image dimensions *m*, that is, the number of rows is less than the number of columns in matrix **D**, the linear equation *y* = **D** *x* is underdetermined. When the number of rows is more than the number of column rows in matrix **D**, the equation *y* = **D** *x* is a nonunderdetermined equation with a unique solution, and we cannot get the sparse solution. Therefore, the use of stOMP method to classify will not achieve a satisfying recognition rate.

As can be seen from the test results, the proportion of feature dimension obtained after dimension reduction for training samples in the number of training samples will affect the final recognition result. When the training sample is 600 and the feature dimension is 72, the recognition rate is 88.5%, which is a relatively high recognition result. When feature dimension comes to 120 and 168, the recognition rates are 88.9% and 88.9%, respectively, which means that the growth is very slow, indicating that the performance of the algorithm has almost reached the limit at this time. When the feature dimension comes to 210 and 288, recognition begins to draw dramatically. The results show that the feature dimension of 70 is sufficient for sparse reconstruction. Further, the best effect recognition rate appears when the feature dimension is about 120.

**Figure 8:**
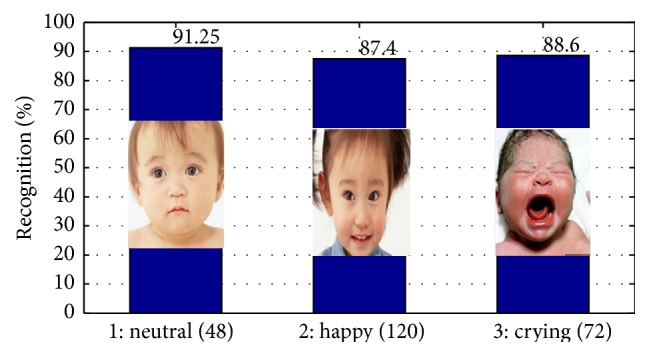
Expression recognition result for three expressions when training sample is 700 (for each expression, training samples and feature dimensions that perform the best are selected).

Meanwhile, in addition to crying expression recognition, we also carry on neutral and happy expression testing. For each expression, we have adopted training samples and feature dimensions that perform the best, as shown in Figure 8. In Figure 8, we can see that, for infants and children expression recognition, the neutral recognition rate is the highest, reaching 91.25%. The false positive rate is quite high for happy and crying expression. On the one hand, these errors are caused due to the self-build database, which lacks image quality compared with standard database. On the other hand, for the specific object infants and children, there is not obvious distinction in terms of happy and crying; therefore, it will be easy to produce confusion with our method.

### 4.2. Comparison of Different Feature Extraction and Dimension Reduction Methods

**Figure 9:**
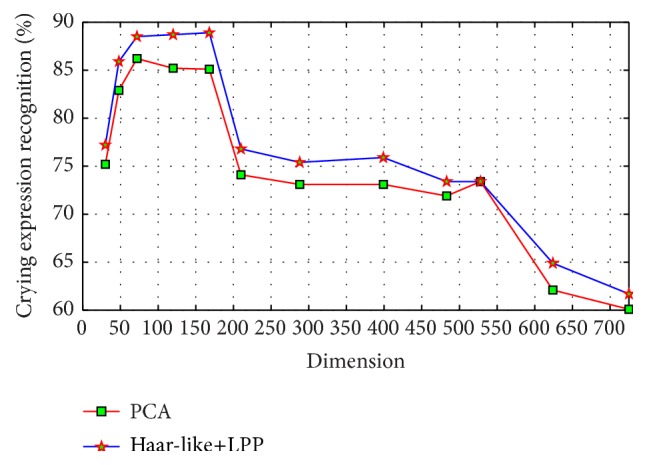
Crying expression recognition results with PCA and Haar-like+LPP methods when the number of training samples is 600.

Use Haar-like+LPP and PCA method to test the sensitivity of our facial expression recognition method to different feature extraction and dimension reduction methods. Take the crying expression recognition; for example, when the number of training samples is 600, recognition result by two feature extraction methods is shown in Figure 9. We can see from Figure 9 that either Haar-like+LPP or PCA can produce a satisfying result, and the highest recognition rate is 88.9% and 86.2%, respectively, with the former a little higher than the latter. This shows that our proposed method is not very sensitive to feature extraction and dimension reduction methods, and the key point is the proportion of feature dimension in the number of training samples.

### 4.3. Influence by Noise

**Figure 10:**
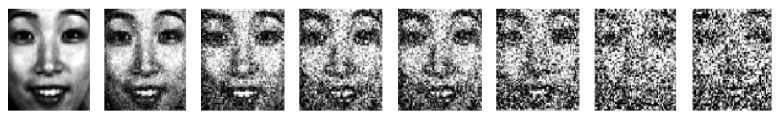
Images after being added to noise (variance from left to right is 0, 0.01, 0.05, 0.1, 0.2, 0.3, 0.4, and 0.5).

To test the algorithm's robustness to noise, the whole test face is added Gaussian random noise, with the steps of variable variance 0.01 and increment from 0 to 0.5. JAFFE database is taken, and parts of the images with noises are shown in Figure 10. We can see that expression images become blurred with the increase of noise variance. When the variance is 0.4, human eyes can hardly distinguish the expression class.

**Figure 11:**
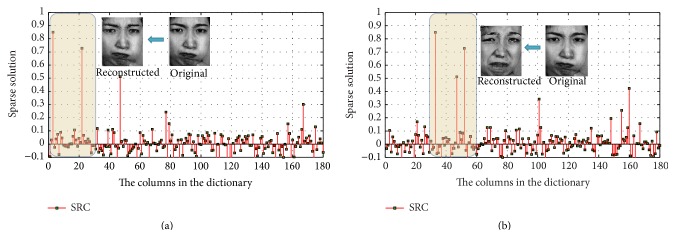
Sparse solution with different noise variances: (a) 0.1; (b) 0.3.

When the noise variance increases, the sparsity gets worse, which leads to a significant decline in the rate of recognition accuracy. Add to the test face random Gaussian noise of zero mean and variable variances 0.1 and 0.3, respectively. Six kinds of expression are selected, 180 in total number, with 30 for each type of expression. The training samples are in a sequence of anger, disgust, surprise, neutral, sadness, and fear. Obviously, the 1–30 columns belong to angry expression, the 31–61 columns belong to disgust expression, and so on. The obtained sparse solutions by SRC with Gaussian noises of variance 0.1 and variance 0.3 are shown in Figures 11(a) and 11(b), respectively.

We find in Figure 11(a) that the maximum sparse solution corresponds to angry expression image, but it corresponds to disgust expression image in Figure 11(b), which is a wrong recognition. With the adding of Gaussian noise, nonzero elements of sparse solution gradually increase, where some solutions are with large values. The sparsity of the solution decreases and affects the expression classification. This shows that the SRC method is not capable of processing facial expression recognition with noises.

**Figure 12:**
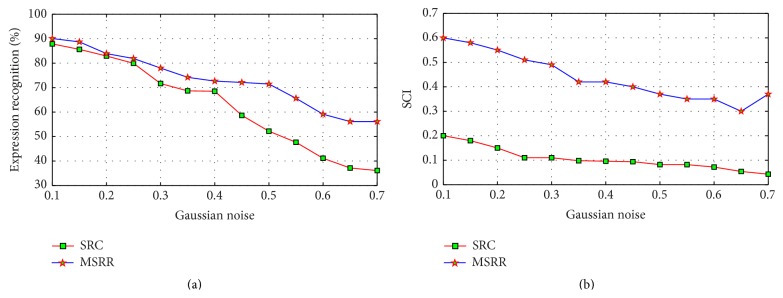
Recognition effect with different variance: (a) recognition rate; (b) SCI.

Then, we repeat the above experiment using modified sparse facial expression recognition method and compare our method with SRC method under different noise variances, as shown in Figure 12.

As can be seen from Figure 12(a), the recognition performances of SRC and MSRR decline with the increase of noise variance. Compared to SRC, the method in this paper has certain robustness to noise, where parameter *γ* plays a role to some extent. In general, the greater the noise is, the greater *γ* is. With the increase of Gaussian noise variance, the extent of decline rate of SRC is greater than that of our method. When the variance is 0.5, recognition rate of SRC is only 52.19%, while the recognition rate of MSRR is 71.58%. In the range of 0 to 0.5 in variance, our method is relatively smooth, with the recognition rate about over 71%.

Compute Sparse Concentration Index (SCI) of sparse coefficient for each recognition, as a measure of sparsity. Average indexes of SCI obtained by 35 faces under different variances are in Figure 12(b). Figure 12(b) shows that, in JAFFE database, with the increase of Gaussian noise variance, SRC's SCI falls faster than the method described in this paper. SCI of our method can maintain 0.3 or more, which verifies that it can weaken noise and improve sparse classification.

Compared to the traditional SRC expression recognition, MSRR methods in this paper contain block training processing, reducing discrimination dictionary error, so as to improve the recognition rate of facial expression. Add noise suppression components during solving sparse solution process, so as to enhance the robustness of sparse representation classification. Therefore, when the noise variance is relatively high, there is still a good recognition rate and sparsity.

### 4.4. Algorithm Comparison

Select dataset 1 and dataset 2 to carry out experiments, and use SVM, RVM, and MSRR algorithm for classification.

(1) Select dataset 1 to make person-dependent face experiments. This set of experiments is to examine the performance of each algorithm immured from outer influence. We select randomly one image per person per expression from CK database, and the rest are for training samples. The total number for testing samples is 178, and that for training samples is 1050, where it is 150 for each face expression samples.

**Figure 13:**
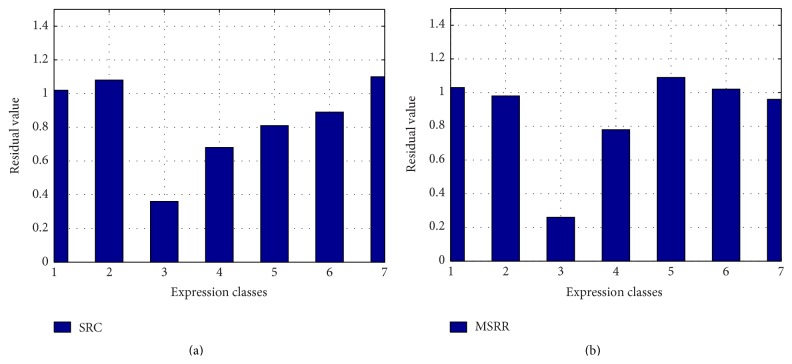
Residual values of SRC and MSRR: (a) SRC; (b) MSRR.

**Figure 14:**
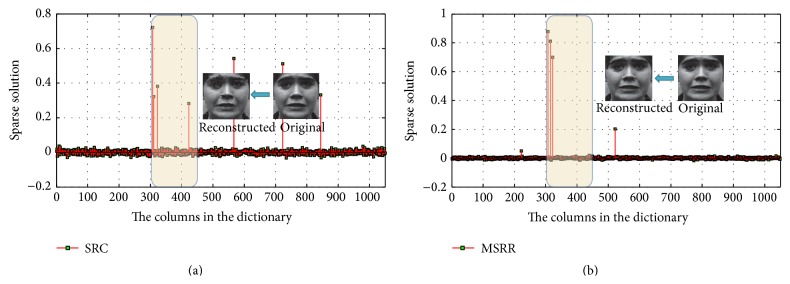
Sparse solution of SRC and MSRR: (a) SRC; (b) MSRR, where the *x* coordinates in (a) and (b) correspond to original images and dictionary atoms, respectively.

To analyze the sparsity of solutions by MSRR, we take the face with fear expression as test face. The recognition results by SRC and MSRR are in Figures 13 and 14, respectively, where the numbers 1–7 represent anger, disgust, fear, happiness, neutral, sadness, and surprise, respectively.

As can be seen from Figure 13, residual value in the third bar is the lowest either in SRC or in MSRR, which can determine that the expression is fear. The difference between the smallest residual value and the second smallest residual value of MSRR is larger than that of SRC, which means that difference between classes in MSRR is relatively significant. We can see from Figure 14 that there are many larger nonzero values widely distributed in SRC, but less nonzero values in MSRR. In other words, the sparsity of SRC is not quite satisfying compared with MSRR solution. The reconstructed image is better for our method than SRC. Our method can improve the recognition rate and sparsity by optimizing overcomplete dictionary, which contains a wealth of information. We also use SVM and RVM for classification, and the result of each classification results is shown in Table 1.

Where the time cost of the proposed method is 49 m, SVM costs a total of 1.35 h and RVM training plus classification costs a total of 2.10 h. We can see that the computation time after optimization by our method is reduced, and the time-consuming part of our method is the dictionary learning stage; the classification time costs short time due to selecting more than a single element in each iteration.

**Figure 15:**
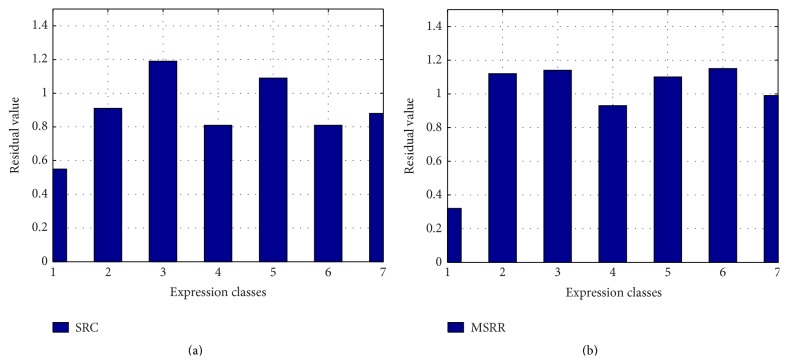
Residual values of SRC and MSRR: (a) SRC; (b) MSRR.

**Figure 16:**
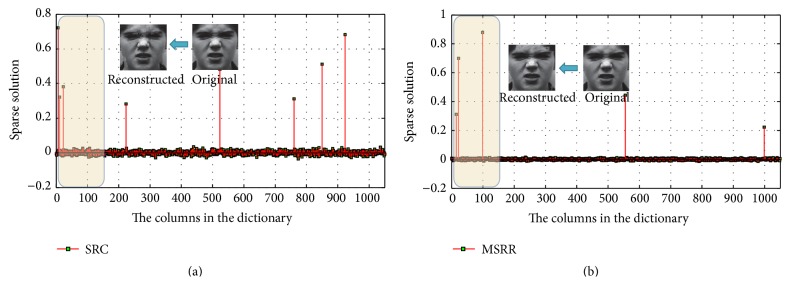
Sparse solution of SRC and MSRR: (a) SRC; (b) MSRR, where the *x* coordinates in (a) and (b) correspond to original images and dictionary atoms, respectively.

(2) Choose dataset 2; person-independent facial expression recognition is more difficult than person-dependent case. In person-independent facial expression recognition, expression information is susceptible to be interfered by facial feature information, leading to a unsatisfying recognition rate. The CK database is divided into 18 parts, where 17 parts are taken for training and the rest for testing. Make sure that every sample can be as test target and at the same time is not in the training sample. The results are achieved by averaging the total 18 recognition results. Take the expression anger as test object, and get the final result by SRC and MSRR, which is shown in Figures 15 and 16, respectively.

In Figure 15, the results for SRC and MSRR are on the left and right, respectively. For MSRR, we can accurately determine the test face expression as angry expression from the smallest residual value bar. In addition, we can see from Figure 16 that the sparse solution in SRC has larger values on some of the columns in the corresponding dictionary, while there are relatively less number of larger nonzero values in MSRR. That is to say, the sparsity is better than SRC method.

**Figure 17:**
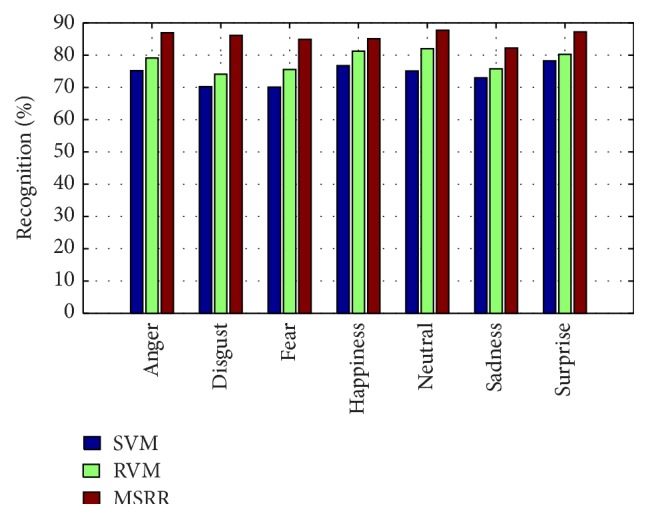
Person-independent facial expression recognition accuracy comparison between MSRR, RVM, and SVM.

We take the use of SVM and RVM for comparison, and the classification result is shown in Figure 17. We can see that classification accuracy of every algorithm is not satisfying because of too many unknown samples. MSRR algorithm suffers less influence of unknown samples compared with SVM and RVM algorithm. For MSRR method, the expression recognition rate for person-independent cases is lower than person-dependent cases, along with the SCI index.

## 5. Summary and Discussion

In this paper, we study expression recognition by sparse representation method. Firstly, we use Haar-like+LPP to extract feature and reduce dimension. Add block dictionary training mode to LC-K-SVD instead of adopting directly the dictionary from samples. Use stOMP on the classification stage to speed up the convergence rate of the traditional OMP and a dynamic regularization factor to suppress noises and enhance accuracy. In typical and self-built database, we select part of the samples for training and testing, to verify the sensitivity to different training samples, different feature dimensions, different feature extraction, and dimension reduction methods, noises, so as to verify the feasibleness and effectiveness of proposed sparse representation classification method. Further, our method is compared with SVM and RVM to analyze the effect of the recognition algorithm and time complexity. Experimental results show that when the sample size is 600 and the extracted features dimension is about 120, the method can achieve best reconstruction and get a better recognition rate. In addition, the proposed recognition method is not very sensitive to feature extraction methods (Haar-like+LPP or PCA). In case there is feasible feature space dimension, we can get satisfying sparse solution. The proposed method can suppress noises to a certain extent due to the use of dynamic regularization factor but perform not quite well for person-independent facial expression recognition. The above experiments illustrate the feasibility of our sparse representation method, which can be better applied to facial expression analysis and has its own advantages in certain aspects.

## Figures and Tables

**Table 1:** Result for each classification algorithm (%).

Method	Expression						
	Class 1	Class 2	Class 3	Class 4	Class 5	Class 6	Class 7
SVM	78.71	72.07	71.22	79.00	77.85	76.00	81.56
RVM	81.20	76.98	76.88	82.53	83.35	77.44	82.64
MSRR	88.88	86.85	86.14	87.45	88.15	83.02	89.99
